# A Case of *Brucella* Endocarditis in Association with Subclavian Artery Thrombosis

**DOI:** 10.1155/2012/581489

**Published:** 2012-07-10

**Authors:** Claudia Colomba, Lucia Siracusa, Raffaella Rubino, Marcello Trizzino, Francesco Scarlata, Claudia Imburgia, Lucina Titone

**Affiliations:** Dipartimento di Scienze della Promozione della Salute G. D'Alessandro, Sezione di Malattie Infettive, Università di Palermo, 90127 Palermo, Italy

## Abstract

Brucellosis is a common zoonosis, endemic in Mediterranean countries, and caused by bacteria of *Brucella* genus. Brucellosis is a systemic infection and the clinical presentation varies widely from asymptomatic and mild to severe disease. Cardiovascular complications are extremely rare. We present a case of arterial thrombosis in a previously healthy young patient with *Brucella* endocarditis. Careful attention must be paid to any sign or symptom of thrombosis in patients affected by brucellosis, regardless of the presence of endocarditis and cardiovascular risk factors.

## 1. Introduction

Brucellosis is a common zoonosis, endemic in Mediterranean countries, and caused by bacteria of *Brucella* genus. Infected animals and their products, particularly unpasteurized milk and dairy products, are common sources of infection. Nearly 200 cases have been recently reported every year in Italy, more than half in Sicily [1, 2]. The clinical presentation varies widely from asymptomatic and mild to severe disease. Brucellosis is a systemic infection: spleen, liver, and bone marrow, rich in reticuloendothelial cells, are the most frequently involved sites. Cardiovascular, gastrointestinal, osteoarticular, urogenital, haematological, and neurological complications may arise [3]. Cardiovascular complications occur in less than 2 percent of cases, but the 80 percent of deaths from this disease are due to the onset of endocarditis [4, 5]. The vascular involvement is quite rare and includes arterial aneurysm formation in different vessels, peripheral arteritis, arterial thrombosis, cutaneous vasculitis, deep venous thrombosis, and cerebral vein thrombosis [6–11].

We present here a case of subclavian artery thrombosis in a patient with *Brucella* endocarditis.

## 2. Case Presentation

In March 2011, a 35-year-old man was admitted to the Institute of Infectious Diseases, “Paolo Giaccone” University Polyclinic in Palermo, because of intermittent fever, arthralgias, and sweating for two months. He also reported a one-week history of intermittent pain in his left arm. He was employed at a slaughterhouse and regularly ate unpasteurized cheese. On admission, physical examination showed hepatosplenomegaly and on cardiac auscultation a 3/6 systolic murmur was heard all over the precordium. Laboratory test results revealed an increase in aspartate aminotransferase (98 U/L, upper normal limit 37 U/L), alanine aminotransferase (200 U/L, upper normal limit 41 U/L), and C-reactive protein (1.6 mg/dL, upper normal limit 1 mg/dL). Electrocardiogram (ECG) showed normal sinus rhythm. A transthoracic echocardiogram was performed and showed mild aortic and mitral insufficiency. A rapid agglutination test (Rosa Bengala Test) and Wright's serum agglutination were performed and they were both positive.

*Brucella melitensis *was isolated from blood culture. The patient was treated with oral doxycycline (100 mg twice a day for 6 weeks) and intravenously (IV) rifampin (450 mg twice a day for 3 weeks). Oral rifampin at the same dose was administered for a further three weeks after hospital discharge. Fifteen days after the end of treatment, the patient had a recurrence of fever, asthenia, and hiporexia. The physical examination did not show remarkable changes. Echocardiogram showed an aortic valve vegetation (1.4 × 0.9 in diameter) with eccentric jet of insufficiency, mitral valve with minimal prolapse of the anterior flap, mild mitral, and tricuspid insufficiency. Laboratory test results revealed a total white blood count of 3830 cells/mm^3^, haemoglobin 11.8 g/dL (normal range 13–17 g/dL), platelet 163000 cells/mm^3^ (normal range 150–400 × 10^3^/mm^3^), erythrocyte sedimentation rate 6 mm/h (upper normal limit 15), C-reactive protein 4.68 mg/dL, and lactate dehydrogenase 634 U/L (normal range 240–480 U/L).

*Brucella melitensis* was isolated from blood culture. PCR assay on blood for detection of *Brucella* was positive. A treatment with IV rifampin (450 mg twice a day) and oral doxycycline (100 mg twice a day) was resumed and IV amikacin (1 gr once a day) was started. Nine days after admission, the patient experienced acute left upper limb pain. Decreased pulses and decreased skin temperature of the arm were noticed. Echo Doppler showed thrombosis of the left subclavian artery. The CT scan showed axillary artery filling defect with side calcification. Heparin (25000 U/24 h) was administered for ten days and the patient underwent an aortic valve replacement surgery with prosthesis implantation. The treatment with IV rifampin (450 mg twice a day), IV amikacin (1 gr once a day), and oral doxycycline (100 mg twice a day) was stopped after 45 days. Oral rifampin and doxycycline, at the same dose, were administered for further 45 days with complete resolution of symptoms. Blood cultures became negative after ten days of triple therapy.

## 3. Discussion

*Brucella* is able to infect endothelial cells and to induce a powerful inflammatory response, leading to possible cardiovascular complications [12]. Endocarditis of native or, more often, prosthetic valves is the most common presentation of cardiovascular involvement [13, 14]. Vascular complications, extremely rare, include arterial aneurysm in any blood vessels (aorta, brachial, tibioperoneal and cerebral arteries) and venous and arterial thrombosis with or without underlying endocarditis [6–11]. The inflammatory response and the immune reaction to *Brucella* antigens in the vessel wall are suggested mechanisms for pathogenesis of vascular involvement. Endothelial activation in response to *Brucella* infection, with upregulation of adhesion molecules and secretion of proinflammatory chemokines, may play an important role [12].

It is not clear if *Brucella* endocarditis in our patient has been an early presentation (on admission cardiac auscultation revealed a 3/6 systolic murmur all over the precordium) or a sign of relapse after 15 days. If endocarditis has been a relapse, doxycycline plus rifampicin administered during the first hospitalization has not been an effective regimen. There is evidence that classical therapeutic regimen (doxycycline for 6 weeks and streptomycin for 3 weeks) is slightly more efficacious in preventing relapses compared to doxycycline (for 6 weeks) and rifampicin (for 6 weeks) [15].

On the other hand, subclavian artery thrombosis can be considered as a complication of endocarditis or as a primary vascular complication due to the direct localization of the microorganism in the subclavian artery. The patient actually complained about left upper limb pain during the first hospitalization and this symptom was at that time interpreted as arthralgias, but thrombosis had probably already started.

## 4. Conclusions

Our patient was a young and otherwise healthy man without cardiovascular risk factors, with an insidious presentation of the illness. We think that it is mandatory to perform an accurate cardiac physical examination in patients with brucellosis at admission and during the followup, especially in patients not responding to therapy or with relapse. Careful attention must also be paid to any sign or symptom of thrombosis in patients affected by brucellosis, regardless of the presence of endocarditis and cardiovascular risk factors.
